# A robustness metric for biological data clustering algorithms

**DOI:** 10.1186/s12859-019-3089-6

**Published:** 2019-12-24

**Authors:** Yuping Lu, Charles A. Phillips, Michael A. Langston

**Affiliations:** Department of Electrical Engineering and Computer Science, University of Tennessee, Knoxville, 37996 TN USA

**Keywords:** Robustness, Clustering algorithms, Paraclique

## Abstract

**Background:**

Cluster analysis is a core task in modern data-centric computation. Algorithmic choice is driven by factors such as data size and heterogeneity, the similarity measures employed, and the type of clusters sought. Familiarity and mere preference often play a significant role as well. Comparisons between clustering algorithms tend to focus on cluster quality. Such comparisons are complicated by the fact that algorithms often have multiple settings that can affect the clusters produced. Such a setting may represent, for example, a preset variable, a parameter of interest, or various sorts of initial assignments. A question of interest then is this: to what degree do the clusters produced vary as setting values change?

**Results:**

This work introduces a new metric, termed simply “robustness”, designed to answer that question. Robustness is an easily-interpretable measure of the propensity of a clustering algorithm to maintain output coherence over a range of settings. The robustness of eleven popular clustering algorithms is evaluated over some two dozen publicly available mRNA expression microarray datasets. Given their straightforwardness and predictability, hierarchical methods generally exhibited the highest robustness on most datasets. Of the more complex strategies, the paraclique algorithm yielded consistently higher robustness than other algorithms tested, approaching and even surpassing hierarchical methods on several datasets. Other techniques exhibited mixed robustness, with no clear distinction between them.

**Conclusions:**

Robustness provides a simple and intuitive measure of the stability and predictability of a clustering algorithm. It can be a useful tool to aid both in algorithm selection and in deciding how much effort to devote to parameter tuning.

## Background

Clustering algorithms are generally used to classify a set of objects into subsets using some measure of similarity between each object pair. Comparisons between clustering algorithms typically focus on the quality of clusters produced, as measured against either a known classification scheme or against some theoretical standards [1–3]. In the former case, varying criteria for what constitutes a meritorious cluster are often applied, employing domain-specific knowledge such as ontological enrichment [4, 5], geographical alignment [6] or legacy delineation [7]. In the latter case, statistical quality metrics are most often used, with cluster density something of a gold standard. Examples include modularity [8], which measures the density of connections within clusters versus density of connections between clusters, clustering coefficient [9, 10], which gives the proportion of triplets for which transitivity holds, and silhouette coefficient [11], which is based on how similar a node is to its own cluster as compared to other clusters. Additional metrics include the adjusted rand index [12], homogeneity [13], completeness [14], V-measure [15], and adjusted mutual information [16]. No single algorithm is of course likely to perform best over every metric.

In this paper, we consider algorithmic comparisons from another perspective. Rather than attempt to measure the quality or correctness of the clusters themselves, we focus instead on the sensitivity of an algorithm’s clusters to changes in its various settings. The metric we introduce, which we term “robustness”, provides a relatively simple measure of a clustering algorithm’s stability over a range of these settings. We note that robustness should not be confused with other clustering appraisals such as correctness or resistance to noise, which are studied elsewhere in the literature. And while it might seem tempting to try to combine multiple notions, such as accuracy and robustness, into some single metric, the resultant analysis is fraught with complexity and well beyond the scope of this work.

In order to demonstrate the utility of robustness, we chose transcriptomic data publicly available from the Gene Expression Omnibus (GEO) [17]. This is a relevant and logical choice given current technology because of gene co-expression data’s ready abundance, availability and standardized format, and because clustering of this sort of data is such an overwhelmingly common task in the research community’s quest to discover and delineate putative molecular response networks.

## Methods

### Algorithms

Clustering algorithms typically have one or more adjustable settings. For instance, such a setting may denote a preset variable, a relevant parameter, or sets of initial assignments. Sometimes the only setting available is the number of clusters desired. To make the scope of this work manageable, and to keep comparisons as equitable as possible, we only consider algorithms that produce non-overlapping clusters, and that are unsupervised, in the sense that classes into which objects are clustered are not defined in advance. (We deviate from this very slightly in the case of Nearest Neighbor Networks (NNN) [18], which allows a pair of clusters to share a single element.) For each method considered we selected a range of settings commonly used in practice.

Different algorithms may produce (sometimes vastly) different clusters, as may different settings of the same algorithm. In a previous comparison of genome-scale clustering algorithms [1], we focused on cluster enrichment, using Jaccard similarity with known Gene Ontology (GO) and Kyoto Encyclopedia of Genes and Genomes (KEGG) annotation sets as a measure of cluster quality. In that study, graph-theoretical methods outperformed conventional methods by a wide margin. A natural question then is whether something along the same line may hold for robustness.

### Robustness

We seek to define a measure of robustness that can provide a single, easily-interpretable metric that captures the tendency of a clustering algorithm to keep pairs of objects together over a range of settings. Indeed, each algorithm may have its own optimum settings. We did not try to isolate such settings, but rather to measure an algorithm’s sensitivity to parameter variations. Let us consider the results of a single clustering algorithm (ALG). If in any run ALG assigns a pair *P* of objects to at least one cluster, then we define *P*’s robustness to be the proportion of clustering runs in which *P* appears together in any cluster. Thus, for example, if genes *A* and *B* appear together (in any cluster) in 17 of 23 clustering runs, then the score for that pair is 17 / 23 = 0.7391. We extend this from *P* to ALG by defining ALG’s robustness, *R*, as the average score of all such candidates for *P*. In this fashion, robustness is measured for one algorithm and for one dataset, but over multiple runs (setting values).

Formally, we therefore set *R*=*t*/(*dr*), where *t* denotes the total number of (not necessarily distinct) pairs of objects that appear together in some cluster summed over all runs, *d* represents the number of distinct pairs of objects that appear together in some cluster produced by some run, and *r* is the number of times the clustering algorithm was run, each run using a different value for some setting of interest. In other words, robustness is the proportion of clustering runs in which a pair of entities appears together in some cluster, given that they appear together in a cluster in at least one run, averaged over all such pairs. *R* thus lies in the interval (0, 1] and, when all else is equal, we seek algorithms with *R* values as high as possible. Note that the effect of a pair appearing (or failing to appear) in a cluster is typically minor as it only reduces by one the denominator in the above formula. In order to compare robustness values fairly, we were careful to select a range of values that produced clusters of the same scale. The number of clusters was not a consideration, except of course for algorithms such as k-means where the number of clusters is itself the parameter being varied.

**Fig. 1:**
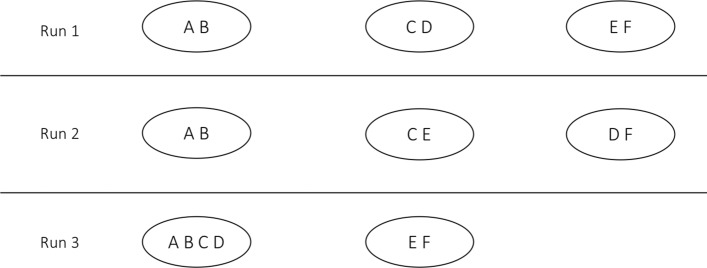
Clusters produced by three runs of a clustering algorithm

We illustrate the notion of robustness with an elementary example based on three runs of some arbitrary clustering algorithm. As shown in Fig. 1, pair (A,B) appears in some cluster in all three runs. Its robustness score is therefore 3/3. Pair (C,D), on the other hand, appears in some cluster in only two of three runs. Its score is thus 2/3. Robustness scores for all pairs that appear in at least one cluster are as follows: (A,B): 3/3; (A,C): 1/3; (A,D): 1/3; (B,C): 1/3; (B,D): 1/3; (C,D): 2/3; (C,E): 1/3; (D,F): 1/3; and (E,F): 2/3. We now simply average these scores to compute *R*, making the robustness of the algorithm that produced these clusters 0.481.

We tested several sorts of clustering algorithms, from conventional hierarchical clustering [19], to partitioning methods such as k-means [20] and Quality Threshold Clustering (QT Clustering) [21], to graph-based methods such as paraclique [22, 23], CLuster Identification via Connectivity Kernels (CLICK) [24], NNN [18] and Weighted Correlation Network Analysis (WGCNA) [25]. We also included Self-Organizing Maps (SOM) [26], a neural network method. Hierarchical clustering assigns items to clusters using a measure of similarity between clusters. Assignments are irrevocable; once an item has been placed in a cluster, it will remain in that cluster. Hierarchical clustering generally comes in two variants: bottom-up (agglomerative), which starts with size one clusters and iteratively combines clusters until only one is left, and top-down, which begins with all genes in one cluster, and then iteratively divides clusters until all clusters are size one. Agglomerative clustering is the simpler and more popular of the two, needing only a linkage criterion to compute cluster similarity. We therefore tested the agglomerative approach with four such criteria: average linkage [27], complete linkage [28], McQuitty [29], and Ward [30].

Graph-based methods model items as vertices, with edges between items determined based again on some sort of similarity measure. To create graphs for transcriptomic data on which to run the paraclique method, we constructed co-expression networks as described in [31]. Genes were thus represented by vertices, while edges were weighted by Pearson product-moment correlation coefficients. A threshold was then applied to the network, so that an edge was retained if and only if its weight was at or above this threshold. In some circles, it has been fashionable to choose an arbitrary threshold, for example 0.85, based on previous experience [32–34]. We prefer a more mathematical and unbiased treatment based on spectral graph theory, whereby eigenvalues are computed over a range of potential thresholds, with the final threshold set using inflection points in network topology [35]. After thresholding, the paraclique method employs clique to help find extremely densely-connected subgraphs, but ones that may be missing a small number of edges [22, 23]. To generate such a cluster, paraclique isolates a maximum clique, then uses a controlled strategy to combine other vertices with high connectivity. Paraclique vertices are then removed from the graph, and the process repeated to find subsequent paraclique clusters. CLICK uses a graph-based statistical method to identify kernels and then expands them into full clusters with several heuristic approaches [24]. NNN, like paraclique, depends upon finding cliques, but only cliques of a specified (typically small) size. It edits a graph by connecting each vertex only to the k most similar other vertices according to some metric such as Pearson correlation, where k is a user-selected value. NNN merges overlapping cliques in the resulting graph to form an initial set of networks. It then divides the preliminary network at any existing articulation points, and ensures that no cluster is larger than half the number of input vertices. WGCNA operates on weighted networks using a soft threshold, raising the similarity matrix to a user-selected power in order to calculate extended adjacencies [25]. It then identifies gene modules using average linkage hierarchical clustering and dynamic tree cut methods. K-means clustering [20, 36] randomly selects k centroids and assigns genes to the nearest centroid, iteratively reassigning and recalculating centroids until it converges. QT Clustering is a method developed specifically for gene expression data [21]. It builds a cluster for each gene, outputs the largest cluster, then removes these genes and repeats the process until no genes remain. SOM is a machine learning approach that groups genes using unsupervised neural networks. SOM repeatedly assigns genes to the most similar node until the algorithm converges [26].

In all, we tested four hierarchical methods, four graph-based methods, two partitioning methods, and one neural network method. We used publicly available versions of each technique. Most are available in R [37]. Table 1 provides a summary, along with the setting we varied for each algorithm.

**Table 1:** Clustering methods tested for robustness

Algorithm	Type	Setting	Implementation
Average	Hierarchical	Number of clusters	R 3.2.3
Complete	Hierarchical	Number of clusters	R 3.2.3
Mcquitty	Hierarchical	Number of clusters	R 3.2.3
Ward	Hierarchical	Number of clusters	R 3.2.3
CLICK	Graph-based	Cluster homogeneity	Expander4
NNN	Graph-based	Min neighborhood size	Java
Paraclique	Graph-based	Starting clique	C++
WGCNA	Graph-based	Power	R 3.2.3
K-means	Partitioning	Number of clusters	R 3.2.3
QT Clustering	Partitioning	Max cluster diameter	R 3.2.3
SOM	Neural network	Grid type/size	R 3.2.3

### Data

In previous work [1] we used *Saccharomyces cerevisiae* data from [38] to test cluster quality. In this paper, we expand the test suite to 24 gene co-expression datasets from GEO, including the species *Drosophila melanogaster*, *Escherichia coli*, *Mus musculus* and *Penicillium chrysogenum*. Data from these organisms have been well-studied and annotated. All data are log2 transformed. Table 2 provides an overview of these datasets, along with the threshold selected using the aforementioned spectral techniques.

**Table 2:** Gene expression datasets tested in this study

Dataset	Organism	Threshold	Edges	Vertices
GDS516	Drosophila melanogaster	0.89	3980	195322
GDS2485	Drosophila melanogaster	0.91	4604	30412
GDS2504	Drosophila melanogaster	0.81	7888	191715
GDS2674	Drosophila melanogaster	0.95	3334	5820
GDS1842	Drosophila melanogaster	0.91	2307	4589
GDS653	Drosophila melanogaster	0.95	1688	3368
GDS664	Drosophila melanogaster	0.8	14008	2298635
GDS1399	Escherichia coli	0.95	2880	5614
GDS5160	Escherichia coli	0.94	4826	74819
GDS5162	Escherichia coli	0.95	5038	293061
GDS5010	Mus musculus	0.9	10269	120907
GDS3870	Penicillium chrysogenum	0.94	6826	62431
GDS344	Saccharomyces cerevisiae	0.95	3071	6303
GDS772	Saccharomyces cerevisiae	0.94	1463	3785
GDS777	Saccharomyces cerevisiae	0.91	2244	11916
GDS1013	Saccharomyces cerevisiae	0.81	5312	555852
GDS1103	Saccharomyces cerevisiae	0.95	4215	38139
GDS1534	Saccharomyces cerevisiae	0.8	9335	1470003
GDS1674	Saccharomyces cerevisiae	0.93	3839	11904
GDS2267	Saccharomyces cerevisiae	0.83	4676	302104
GDS2508	Saccharomyces cerevisiae	0.9	3069	10485
GDS2663	Saccharomyces cerevisiae	0.8	9335	2617139
GDS3332	Saccharomyces cerevisiae	0.86	7290	572118
GDS2969	Saccharomyces cerevisiae	0.95	1679	5206

### Comparisons

To compare algorithmic robustness, we altered a common setting for each method as specified in Table 1, selecting a range of values that produced clusters of the same scale. We transformed the myriad of output formats to simple cluster/gene membership lists. We also controlled r, the number of runs (values for each setting), to reduce its influence on our results. Runtime performance was not a consideration, although one algorithm, QT Clustering, never finished on dataset GDS5010, even after two weeks. We did not therefore obtain QT Clustering robustness for that input. The robustness of each algorithm on each dataset was calculated for all runs over the range of settings.

Three algorithms (k-means clustering, hierarchical clustering and SOM) take the desired number of clusters as input. We thus selected this as the most appropriate setting to alter, and tested values from 200 to 300 so as to produce a range of average cluster sizes in line with the other algorithms. For example, hierarchical clustering produces a tree of clusters, and one obtains a list of disjoint clusters by choosing an articulation point in the tree. For SOM, we transformed the number of clusters to grid size. For example, when using 35 as the number of clusters (for dataset GDS344), the grid size was 5*7. We tested five grid sizes and two grid types (rectangular and hexagonal) for each dataset. We applied ten different powers (2, 4, 6, 8, 10, 14, 18, 22, 26 and 30) for WGCNA. For QT Clustering, we picked up ten different maximum cluster diameters from 0.05 to 0.5 with interval 0.05. For NNN, we chose ten different minimum neighborhood sizes ranging from 16 to 25. For CLICK, we applied nine homogeneity values (0.1, 0.2, 0.3, 0.4, 0.5, 0.6, 0.7, 0.8 and 0.9). For paraclique, we created graphs in the usual fashion, by calculating all pairwise correlations and placing edges between pairs correlated at or above a selected threshold. We controlled the number of paracliques generated so that they are in the same scale with other algorithms. We used the choice of maximum clique as the setting to vary. Dataset GDS772, for example, at threshold 0.94, resulted in a graph with nine maximum cliques. And so it was these nine cliques that provided variation. As can be seen from Table 2, over all inputs the threshold selected by spectral methods ranged from 0.8 to 0.95.

## Results

**Fig. 2:**
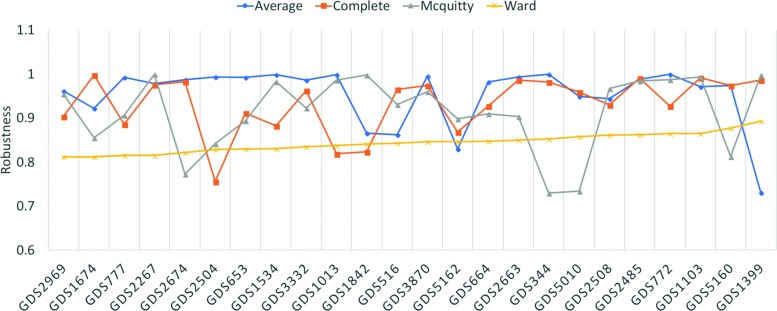
Robustness of four hierarchical algorithms on 24 transcriptomic datasets

**Fig. 3:**
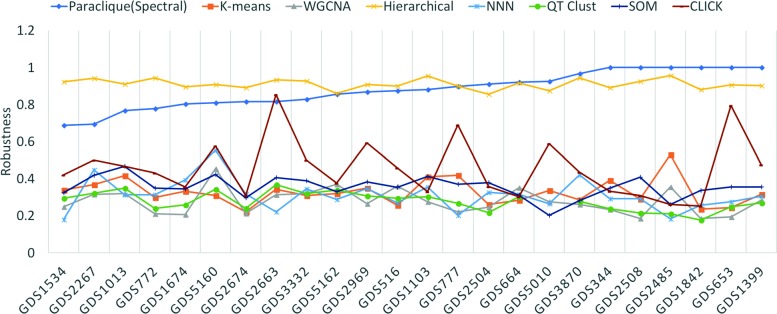
Robustness of all algorithms tested on 24 transcriptomic datasets

**Fig. 4:**
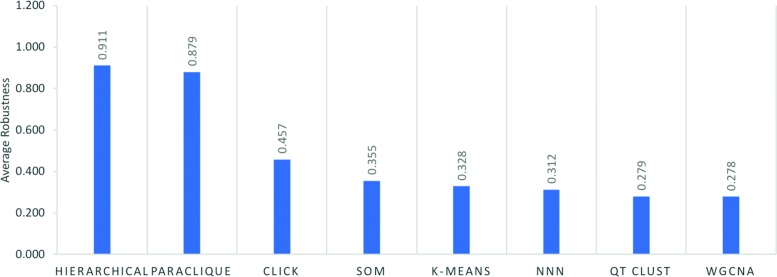
Average robustness of each algorithm

Figure 2 shows robustness results for the four hierarchical algorithms, as tested across the 24 datasets previously described. Because all have robustness above 0.72, we averaged their scores to simplify Fig. 3, which shows robustness results for all algorithms tested. As can be seen from this figure, hierarchical clustering and paraclique exhibit higher robustness than other algorithms. In fact, hierarchical clustering and paraclique have average robustness scores above 0.87, while all others are below 0.5. Figure 4 summarizes the results into an average robustness of each algorithm.

**Fig. 5:**
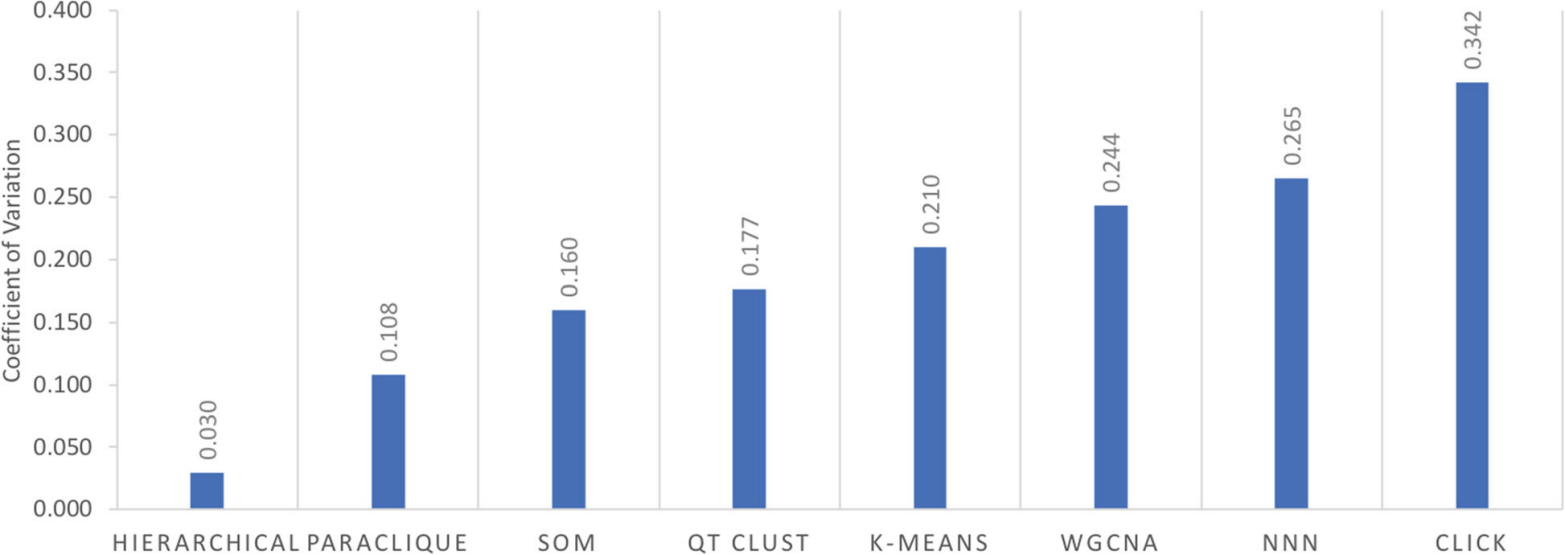
Coefficient of variation of each algorithm

We also calculated the coefficient of variation (CV), the ratio of the standard deviation to the mean, as a measure of the stability of an algorithm’s robustness. Hierarchical clustering exhibits the lowest CV, meaning that its robustness varies little across different datasets, whereas CLICK exhibits the highest CV. See Fig. 5.

## Discussion

It is not unexpected that hierarchical methods display the highest overall robustness. After all, results thereby produced form a hierarchical tree of successively merged clusters, so that varying the number of clusters simply cuts the tree at a different height, while the tree itself does not change. Once a pair of items appears together in some cluster, any decrease in the number of clusters on subsequent runs will continue to place that pair into the same cluster. One might expect similar behavior from WGCNA, since it uses hierarchical clustering to identify modules. Because WGCNA uses soft-power to construct its network, however, the topology of each weighted network changes with different powers, so that item pairs are not at all stable. For k-means, as one alters the number of clusters (and hence centroids), the centroid with which a particular item is associated can change, while not changing an item’s neighbors’ centroids. Thus, items often shift to different clusters as the number of clusters changes. SOM and QT Clustering behave in similar fashion, in that grid size has a large effect on SOM while the partitioning performed by QT Clustering can divide pairs of formerly clustered items. Of the graph-based methods, CLICK and NNN first try to find a base cluster and then absorb other items into it. The absorbed items may change with different settings, affecting the clusters generated. For paraclique, the high robustness with different starting cliques is likely due in part to the fact that many of these cliques have significant overlap [39], at least on transcriptomic data. Many gene pairs may thus be included in a given cluster, no matter which maximum clique is selected. We have also observed quite similar overlap in graphs derived from many diverse types of data, including for example that derived from social and communications networks.

It is probably worth noting how robustness compares to accuracy and sensitivity [40], two popular clustering metrics. Accuracy measures faithfulness to ground truth. We make no assumptions, however, that ground truth is available or that it can even be known. Sensitivity most commonly refers to random noise or outliers. Robustness is not really related to either. A clustering algorithm could be highly sensitive to random noise, for example, and still have either high or low robustness.

This brings us to interpretation. How is the user to make sense of all this information? In our opinion, an algorithm with high robustness is generally preferable whenever it is difficult to determine optimum parameter settings. This is of course because its results are unlikely to vary greatly across an entire range of these settings. As a case in point, if ground truth is largely unknown, or if hierarchical structure is implicit in the data under study, then hierarchical clustering can serve at least as a good starting candidate given its excellent robustness, relative simplicity and intuitive appeal. For more complex clustering tasks, however, we would endorse instead a graph-theoretical method such as paraclique due its solid overall robustness and its much improved potential for biological fidelity [1].

## Conclusions

We have introduced a new clustering metric, termed “robustness”, in an effort to provide the research community with a simple, intuitive and informative measure of the stability and predictability of a clustering algorithm’s behavior. To demonstrate its use, we have employed a suite of transcriptomic datasets as an unbiased testbed for algorithmic variation and evaluation. Widely-available data such as this provides a well-understood basis on which to introduce, explain and illustrate the use of the robustness metric. We hasten to add that robustness can, quite naturally, be applied to virtually any sort of omics data, or in fact to practically any sort of data on which clustering may be performed.

Simple hierarchical clustering displayed the highest overall robustness, due no doubt to the rigidly fixed tree structure of its clusters. Of the more sophisticated methods tested, only paraclique demonstrated similar robustness, thus demonstrating its resilience to the choice of starting maximum clique. In practice, one might expect that selecting such a clique with, say, the highest overall edge weight would be preferable. And certainly that has much intuitive appeal. Nevertheless, our results show that it does not really much seem to matter, at least on data akin to those we’ve employed here.

Open questions abound. Note, for example, that robustness can be applied to virtually any non-overlapping clustering algorithm. All one needs is a reasonable settings range. What then of powerful clustering algorithms like clique? Clique is nonparametric and thus without settings. And one of its core strengths is actually its propensity to produce overlapping clusters on biological data (genes, for example, are very often pleiotropic, and thus likely to belong to multiple clusters). We are studying these and other related questions, and observe that for methods such as clique, in fact for essentially all clustering methods, an alternate notion of robustness might try to capture output predictability as the underlying network is perturbed.

## Data Availability

The datasets used in this paper are available at GEO (https://www.ncbi.nlm.nih.gov/geo/).

## Abbreviations

ALG: A single clustering algorithm
CLICK: Cluster identification via connectivity kernels
CV: Coefficient of variation
GEO: Gene expression omnibus
GO: Gene ontology
KEGG: Kyoto encyclopedia of genes and genomes
NNN: Nearest neighbor networks
QT Clustering: Quality threshold clustering
SOM: Self-organizing maps
WGCNA: Weighted correlation network analysis

## References

[1] Jay JJ, Eblen JD, Zhang Y, Benson M, Perkins AD, Saxton AM, Voy BH, Chesler EJ, Langston MA (2012). A systematic comparison of genome-scale clustering algorithms. BMC Bioinformatics.

[2] Chen G, Jaradat SA, Banerjee N, Tanaka TS, Ko MS, Zhang MQ (2002). Evaluation and comparison of clustering algorithms in analyzing es cell gene expression data. Stat Sin.

[3] Datta S, Datta S. BMC Bioinformatics. 2006; 7(1):397. 10.1186/1471-2105-7-397 PMC1590054 16945146

[4] Subramanian A, Tamayo P, Mootha VK, Mukherjee S, Ebert BL, Gillette MA, Paulovich A, Pomeroy SL, Golub TR, Lander ES (2005). Gene set enrichment analysis: a knowledge-based approach for interpreting genome-wide expression profiles. Proc Natl Acad Sci.

[5] Huang DW, Sherman BT, Tan Q, Kir J, Liu D, Bryant D, Guo Y, Stephens R, Baseler MW, Lane HC, et al. Nucleic Acids Res. 2007; 35(suppl_2):169–75. 10.1093/nar/gkm415 PMC1933169 17576678

[6] De Vries GK, Van Hage WR, Van Someren M. Comparing vessel trajectories using geographical domain knowledge and alignments. In: Data Mining Workshops (ICDMW), 2010 IEEE International Conference On. IEEE: 2010. p. 209–16.

[7] Liu M, Samal A (2002). Cluster validation using legacy delineations. Image Vis Comput.

[8] Newman ME (2006). Modularity and community structure in networks. Proc Natl Acad Sci.

[9] Luce RD, Perry AD (1949). A method of matrix analysis of group structure. Psychometrika.

[10] Wasserman S, Faust K (1994). Social Network Analysis: Methods and Applications. vol. 8.

[11] Rousseeuw PJ (1987). Silhouettes: a graphical aid to the interpretation and validation of cluster analysis. J Comput Appl Math.

[12] Rand WM (1971). Objective criteria for the evaluation of clustering methods. J Am Stat Assoc.

[13] Hansen P, Jaumard B (1997). Cluster analysis and mathematical programming. Math Program.

[14] Hubert L (1973). Min and max hierarchical clustering using asymmetric similarity measures. Psychometrika.

[15] Rosenberg A, Hirschberg J. V-measure: A conditional entropy-based external cluster evaluation measure. EMNLP-CoNLL 2007. 2007:410.

[16] Vinh NX, Epps J, Bailey J. J Mach Learn Res. 2010; 11(Oct):2837–54.

[17] Edgar R, Domrachev M, Lash AE. Nucleic Acids Res. 2002; 30(1):207–10. 10.1093/nar/30.1.207 PMC99122 11752295

[18] Huttenhower C, Flamholz AI, Landis JN, Sahi S, Myers CL, Olszewski KL, Hibbs MA, Siemers NO, Troyanskaya OG, Coller HA. BMC Bioinformatics. 2007; 8(1):250. 10.1186/1471-2105-8-250 PMC1941745 17626636

[19] Johnson SC (1967). Hierarchical clustering schemes. Psychometrika.

[20] Hartigan JA, Wong MA (1979). Algorithm as 136: A k-means clustering algorithm. J R Stat Soc Ser C (Appl Stat).

[21] Heyer LJ, Kruglyak S, Yooseph S (1999). Exploring expression data: identification and analysis of coexpressed genes. Genome Res.

[22] Chesler EJ, Langston MA (2007). Combinatorial genetic regulatory network analysis tools for high throughput transcriptomic data. Systems Biology and Regulatory Genomics.

[23] Hagan RD, Langston MA, Wang K (2016). Lower bounds on paraclique density. Discret Appl Math.

[24] Sharan R, Maron-Katz A, Shamir R (2003). Click and expander: a system for clustering and visualizing gene expression data. Bioinformatics.

[25] Zhang B, Horvath S. A general framework for weighted gene co-expression network analysis. Stat Appl Genet Mol Biol. 2005;4(1). 10.2202/1544-6115.1128 16646834

[26] Tamayo P, Slonim D, Mesirov J, Zhu Q, Kitareewan S, Dmitrovsky E, Lander ES, Golub TR (1999). Interpreting patterns of gene expression with self-organizing maps: methods and application to hematopoietic differentiation. Proc Natl Acad Sci.

[27] Seifoddini HK (1989). Single linkage versus average linkage clustering in machine cells formation applications. Comput Ind Eng.

[28] Dawyndt P, De Meyer H, De Baets B (2005). The complete linkage clustering algorithm revisited. Soft Comput.

[29] McQuitty LL. Educ Psychol Meas. 1966; 26(4):825–31.

[30] Ward Jr JH (1963). Hierarchical grouping to optimize an objective function. J Am Stat Assoc.

[31] Voy BH, Scharff JA, Perkins AD, Saxton AM, Borate B, Chesler EJ, Branstetter LK, Langston MA (2006). Extracting gene networks for low-dose radiation using graph theoretical algorithms. PLoS Comput Biol.

[32] Willems E, Guerrero-Bosagna C, Decuypere E, Janssens S, Buyse J, Buys N, Jensen P, Everaert N (2016). Differential expression of genes and dna methylation associated with prenatal protein undernutrition by albumen removal in an avian model. Sci Rep.

[33] Herrer I, Roselló-Lletí E, Ortega A, Tarazón E, Molina-Navarro MM, Triviño JC, Martínez-Dolz L, Almenar L, Lago F, Sánchez-Lázaro I (2015). Gene expression network analysis reveals new transcriptional regulators as novel factors in human ischemic cardiomyopathy. BMC Med Genomics.

[34] Venu R, Madhav MS, Sreerekha M, Nobuta K, Zhang Y, Carswell P, Boehm MJ, Meyers BC, Korth KL, Wang G-L (2010). Deep and comparative transcriptome analysis of rice plants infested by the beet armyworm (spodoptera exigua) and water weevil (lissorhoptrus oryzophilus). Rice.

[35] Perkins AD, Langston MA. Threshold selection in gene co-expression networks using spectral graph theory techniques. In: BMC Bioinformatics. BioMed Central: 2009. p. 4. 10.1186/1471-2105-10-S11-S4 PMC3152776 19811688

[36] MacQueen J, et al. Some methods for classification and analysis of multivariate observations. In: Proceedings of the Fifth Berkeley Symposium on Mathematical Statistics and Probability, vol. 1. Oakland: 1967. p. 281–97.

[37] R Core Team. R: A Language and Environment for Statistical Computing. Vienna: R Foundation for Statistical Computing; 2017. https://www.R-project.org. Accessed 11 Jul 2017.

[38] Gasch AP, Huang M, Metzner S, Botstein D, Elledge SJ, Brown PO (2001). Genomic expression responses to dna-damaging agents and the regulatory role of the yeast atr homolog mec1p. Mol Biol Cell.

[39] Eblen JD, Phillips CA, Rogers GL, Langston MA (2012). The maximum clique enumeration problem: algorithms, applications, and implementations. BMC Bioinformatics.

[40] Baratloo A, Hosseini M, Negida A, El Ashal G (2015). Part 1: simple definition and calculation of accuracy, sensitivity and specificity. Emergency.

